# College and Hospital Notes

**Published:** 1909-11

**Authors:** 


### COLLEGE AND HOSPITAL NOTES.

Dr. L. Duncan Bulkley, of New York, will give an eleventh series of Clinical Lectures on Diseases of the Skin, in the outpatient hall of the hospital on Wednesday afternoons, commencing November 3, 1909, at 4.15 o'clock. The course will be free to the medical profession.

---

The twenty-fourth annual course of instruction in the Buffalo college of pharmacy began on Wednesday, October 13, 1909. The afternoon was devoted to matriculation of students. The faculty and classes assembled in Alumni Hall, University Building, in the evening.

Two important changes occurred in the faculty. A. P. Sy, M.S., Ph.D., became professor of chemistry, succeeding Professor Hill, and Frank E. Lock, M.D., succeeds Professor Gray in the chair of pharmacognosy.

The department of pharmacy now includes three complete courses of study: pharmacy, leading to the degrees bachelor, master or doctor in pharmacy; chemistry, leading to the degrees of analytical chemist; and foods and drugs, qualifying the student to become inspector of foods and drugs.

---

The Board of Managers of The Children's Hospital, of Buffalo, sent out invitations to physicians to visit and inspect the completed hospital building, 219 Bryant street, on Thursday afternoon, October 7, 1909, from three to six o'clock.

---

A course which will prepare young women to become trained attendants is being given at the Young Women's Christian Association, 19 W. Mohawk street, under the direction of Dr. Maud J. Frye. This course will include a year's work, divided into two terms of fifteen weeks each. The class meets three times each week—Tuesday, Thursday, Saturday, and the course opened October 16, 1909. Tuition, $40.00 a year—payable $20.00 each term.

---

The Queen Alexandra Sanatorium (under Her Majesty's patronage), which is to be opened early next autumn, is destined to rank high in the list of the National Sanatoria of cosmopolitan Davos. But though national, it will be unique in welcoming patients from all parts of the world, and not only from the empire but from the states, as it was founded for the benefit of all English-speaking nationalities, the only qualifications needed being evidence of medical suitability and of inability to meet the heavier cost of treatment at hotels or private institutions.

---

The new isolation hospital of the Salvation Army was dedicated Thursday, October 20, 1909. The hospital is located on Cottage street, Buffalo. Major Davis, who is in charge of the work of the Salvation Army in this city, made a brief address. Others present were the Reverend E. H. Coman, Mrs. Irving Mills and Brigadier Margaret Bovell, the national secretary for rescue work.

The new hospital is for the treatment of persons who come to the attention of the army who are suffering from contagious diseases. In times of epidemic the hospital will be opened to city patients.

---

The state is erecting a tuberculosis hospital or annex at the Soldiers' and Sailors' Home, at Bath. Contracts for the construction work were awarded October 20, 1909. Winfield S. Clough, of Bath, obtaining the contract for constructing the building, at $10,100, and Gould & Nowlen of Bath, secured the contract for installing the plumbing and heating.

---

The Children's Hospital, in Bryant street, Buffalo, a notice of the completion of which is published elsewhere, adds another fine building for hospital purposes in the city. Mrs. Georgie B. Pardee is entitled to a full share of credit for the building. She gave the building and made no reckoning. Many private rooms have been equipped by other philanthropic women, and the hospital will ever stand as a monument to the work of the good women who have contributed to its success.

